# Structure-guided protein engineering of ammonia lyase for efficient synthesis of sterically bulky unnatural amino acids

**DOI:** 10.1186/s40643-021-00456-5

**Published:** 2021-10-19

**Authors:** Zi-Fu Ni, Pei Xu, Min-Hua Zong, Wen-Yong Lou

**Affiliations:** Laboratory of Applied Biocatalysis, School of Food Science and Engineering, South China University of Technology, No. 381 Wushan Road, Guangzhou, 510640 Guangdong China

**Keywords:** Biocatalysis, Methyl aspartate lyase, Substrate specificity, Protein engineering, Solid-phase color screening

## Abstract

Enzymatic asymmetric amination addition is seen as a promising approach for synthesizing amine derivatives, especially unnatural amino acids, which are valuable precursors to fine chemicals and drugs. Despite the broad substrate spectrum of methylaspartate lyase (MAL), some bulky substrates, such as caffeic acid, cannot be effectively accepted. Herein, we report a group of variants structurally derived from *Escherichia coli* O157:H7 MAL (*Ec*MAL). A combined mutagenesis strategy was used to simultaneously redesign the key residues of the entrance tunnel and binding pocket to explore the possibility of accepting bulky substrates with potential application to chiral drug synthesis. Libraries of residues capable of lining the active center of *Ec*MAL were then constructed and screened by an effective activity solid-phase color screening method using tyrosinase as a cascade catalyst system. Activity assays and molecular dynamics studies of the resultant variants showed that the substrate specificity of *Ec*MAL was modified by adjusting the polarity of the binding pocket and the degree of flexibility of the entrance tunnel. Compared to M3, the optimal variant M8 was obtained with a 15-fold increase in catalytic activity. This structure-based protein engineering of *Ec*MAL can be used to open new application directions or to develop practical multi-enzymatic processes for the production of various useful compounds.

**Supplementary Information:**

The online version contains supplementary material available at 10.1186/s40643-021-00456-5.

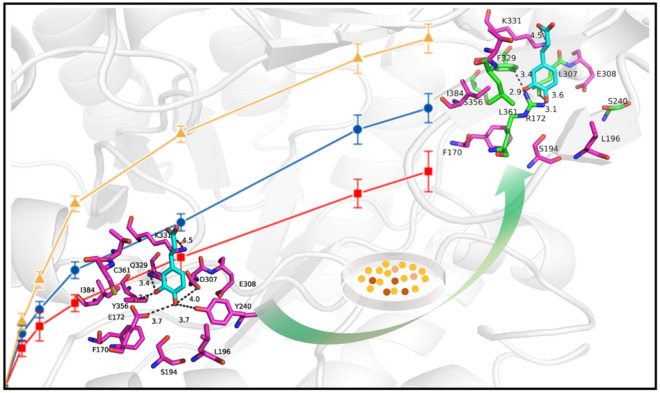

## Introduction

The hydroamination of olefins and carbon–nitrogen bond-forming reactions of unsaturated carboxylic acids offer a vast range of applications in the synthesis of fine chemicals (Raj et al. 2012). However, under classical conditions, the catalysts used are potentially hazardous to the environment. With increasing emphasis on the concept of green, clean, and sustainable chemistry, alternative strategies, such as biological methods, have been proposed for implementing such reactions (Wu et al. 2020). Enzymatic transformation of unsaturated carboxylic acids is an ideal approach for the production of unnatural amino acids, having prominent advantages of economy and sustainability (Zhang et al. 2020a). Six classes of enzymes have been applied for this purpose, as exemplified by ω-transaminase (Mathew et al. 2017; Bea et al. 2011), nitrilase (Yu et al. 2019), amino acid dehydrogenase (Zhang et al. 2015), ammonia lyase (AL) (Zhang et al. 2020b), lipase (Zeng et al. 2018), and tautomerase (Liu et al. 2020). However, based on the principles of atomic-economy and cost-effectiveness, the most straightforward approach is the use of AL as the catalyst to drive the hydroamination of unsaturated carboxylates (Fibriansah et al. 2011). This mild biocatalytic process would offer a welcome alternative for the synthesis of unnatural amino acids and their derivatives.

ALs, belonging to the enolase family, specifically use unsaturated carboxylates as substrates for the hydroamination reaction to generate unnatural amino acids and their derivatives (Turner 2011). Among ALs, MAL has attracted much attention because of its broad substrate specificity and its acceptance of a variety of amines (Leese et al. 2013). Recently, MAL and its variants have been reported to catalyze C–N bond formation in the synthesis of artificial dipeptide sweeteners, showing that they possess broad amine scope in accepting unnatural substrates (Zhang et al. 2020b). However, despite these notable advances, unnatural amino acids based on large-frame organic acids remain largely unstudied. They can be envisaged as playing an important role in the synthesis of many chemicals, such as 3,4-dihydroxycinnamic acid, a precursor in the production of levodopa (l-dopa) (Fordjour et al. 2019).

The potential of enzymes to be modified by directed evolution has attracted much attention in recent years (Kramer et al. 2019). Structure-guided protein engineering has replaced the natural mutation of enzymes as one of the main tools of evolution (Cheng et al. 2018; Renata et al. 2015). For example, Wu and co-workers used structure-based computational enzyme design to successfully convert aspartase into a series of complementary hydroamination biocatalysts showing excellent regio- and enantioselectivity (Li et al. 2018). However, the direction of directed evolution is uncontrollable, so the correct selection of the mutagenesis strategies and the amount of screening verification work are key in determining the ultimate degree of success (Zhou et al. 2016). Screening is the bottleneck of directed evolution, and two strategies are presently in use. In the first, the focus is on developing efficient mutagenesis strategies to generate high-quality libraries to minimize the detection workload (Zhou et al. 2016; Hu et al. 2020; Li et al. 2019). In the second, the aim is to establish high-throughput screening methods, allowing rapid discrimination based on colorimetric or optical changes (Li et al. 2019, 2020).

Our previous studies have already led to the mining of an MAL from *E. coli* O157:H7, which was found to catalyze the hydroamination of short-chain unsaturated acids such as fumaric acid or itaconic acid to the corresponding amino acids with good selectivity. However, a dramatic decrease in reactivity was observed when the substrate side chains increased. Therefore, using structural information as a guide to expand the substrate scope of engineering MALs is the main objective. In this study, we aimed to probe new residues located near the active center and entrance tunnel, which may affect the acceptance of bulky aromatic unsaturated acids. We then further adjusted them to expand the substrate spectrum of MAL.

## Materials and methods

### Chemicals

PrimerSTAR max DNA polymerase was obtained from TaKaRa Biotechnology Co. (Dalian, China). *Escherichia coli* strains BL21 (DE3) and DH5α were bought from TransGen Biotech Co., Ltd. (Beijing, China). All other commercial chemicals were of analytical grade or above, and were purchased from Aladdin (Shanghai, China), Macklin (Shanghai, China), TCI (Tokyo, Japan) or Sigma-Aldrich (St. Louis, USA).

### Homology modeling and molecular docking

The *Ec*MAL homology model was created using SWISS-MODEL (https://swissmodel.expasy.org/), taking MAL from *Clostridium tetanomorphum* (PDB ID:1KKR) as a template (Lambrughi et al. 2019). The modeled protein structure was evaluated through a Ramachandran plot, and the percentage of residues in the allowed region was 98.6%. Molecular docking was performed with Yasara using the default program parameters. The center coordinates of the box were determined by visual molecular dynamics, and the box size was set as 20 Å in each dimension. The docking results were selected according to binding affinities and molecular conformations (Cheng et al. 2018). PyMOL was used to display and analyze the modeled protein (Yuan et al. 2016). Amino acids with low conservation score at the entrance of the active pocket were chosen as hotspots (Yu et al. 2019). CAVER 3.0 (http://www.caver.cz) was used to identify the tunnels present in *Ec*MAL (Zhang et al. 2019).

### PCR-based methods for site mutagenesis and library construction of *Ec*MAL

Site-directed mutagenesis and combinatorial active site saturation testing were conducted by the overlap PCR and megaprimer approach with PrimerSTAR max DNA polymerase. The reaction system had a total volume of 50 μL, comprising 25 μL of PrimerSTAR max polymerase, 0.5 μL (50–100 ng) of template DNA, and 200 μm primers mix (2 μL each), made up to the specified volume with water. PCR amplification protocol for short fragments: 98 °C for 2 min; (98 °C for 30 s, 55 °C for 30 s, 72 °C for 1 min) × 30 cycles; 72 °C for 5 min. PCR products with the above short fragments were then used as primers for mega-PCR: 98 °C for 2 min; (98 °C for 30 s, 60 °C for 30 s, 72 °C for 7 min) × 30 cycles; 72 °C for 10 min. The PCR products were resolved by agarose gel electrophoresis and purified using a Sangon Biotech purification kit. Digestion reaction program: 2 μL of NEB CutSmart buffer and 1 μL of Dpn I were added for every 20 μL of identified PCR product, and the mixture was incubated for 4 h at 37 °C. After thermal inactivation at 80 °C for 20 min, plasmids containing the mutated gene were directly transformed into *E. coli* BL21 (DE3) and then plated on a Luria–Bertani (LB) agar plate with 100 μg/mL of ampicillin.

Multi-site simultaneous mutagenesis is based on specific amino acid selection and primer design, which determines the size and quality of the constructed library. Here, the 19 residues involved in *Ec*MAL were divided into five groups (**A–E**). The short fragments between multiple mutation sites, as primers for the next round of amplification reactions, were then amplified by PCR (Wang et al. 2016). The purified short fragments were used as primers to amplify the whole plasmid PET32a-*Ec*MAL, leading to the final range of plasmids for library generation. After digestion by Dpn I, the PCR products were transformed into *E. coli* BL21 (DE3) cells to create a library for screening. Applying standard transformation procedures, the transformants were spread on HyBond-N membranes, which were then placed on the surface of LB/ampicillin (Amp, 100 μg/mL) agar plates. After incubation overnight at 37 °C, the membranes were transferred to new LB/amp plates with 0.1 mM isopropyl-β-d-thiogalactopyranoside (IPTG) and incubated for 12 h at 30 °C. Each membrane was then transferred to a clean plate and stored at – 20 °C prior to use.

### Screening procedures

Our solid-phase screening procedure is based on the analysis method devised by Turner’s group (Aleku et al. 2017). Membranes containing the clones were repeatedly frozen in liquid N_2_ and thawed four times to lyse the cells. The membranes were then placed on top of a filter paper that was previously loaded with final concentrations of 10 mM screening substrate, 20 mM Mg^2+^, and 500 mM NH_4_Cl in 500 mM Tris–HCl buffer (pH 8.5). After incubation for 6 h at 30 °C, color-changed colonies were picked and inoculated into LB/amp for subsequent activity detection and mutant sequencing comparison. The number of mutants screened is calculated according to the 95% mutation coverage determined by mutation toolbox developed by Reezt’s team (Reetz et al. 2005).

### Protein expression and purification

Wild-type and positive variants of *Ec*MAL were inoculated in 20 mL of LB medium containing 100 μg/mL of ampicillin and incubated at 37 °C and 180 rpm. The overnight cultures were transferred into 500 mL of medium at a 1% inoculum level. When the absorbance of the medium at 600 nm (OD600) reached 0.6–0.8, a final concentration of 0.1 mM IPTG was added, and the cultivation temperature was adjusted to 16 °C to induce protein overexpression. After 20 h, each culture was harvested by centrifuging at 8000*g* for 5 min, and the precipitate was washed twice with 0.9% NaCl solution. The cells were resuspended in buffer A (200 mM Tris–HCl buffer containing 20 mM imidazole, 500 mM NaCl, pH 8.5) and disrupted by ultrasonication. The cell lysates were centrifuged at 10,000*g* for 10 min at 4 °C to remove insoluble debris. The protein supernatant samples were loaded onto a HisTrap Ni–NTA FF column (GE Healthcare, USA) and eluted with buffer B (in which the imidazole concentration was increased to 250 mM). The purified proteins were then further desalted by passage through a desalination column (GE Healthcare, USA). The Bradford method was used to determine the protein concentration.

### Specific activity and enantioselectivity assays of the wild-type and mutant enzymes

The kinetic parameters of *Ec*MAL were first measured with varying concentrations of **1a** (0.25–10 mM) and **1b** (0.2–10 mM) as substrates by detecting the initial reaction rate of the protein and curve-fitting according to the Michaelis–Menten equation. The specific activities and stereoselectivities of the EcMAL mutants toward **1a**, **1b**, and **1c** were then measured using purified enzymes. The reactions were carried out as follows: in a reaction system with a total volume of 2 mL, substrate **1a** (10 mM), **1b** (10 mM), or **1c** (5 mM) in dimethyl sulfoxide (0.5 mL) and the purified enzyme were mixed with Tris–HCl (500 mM, pH 8.5). The mixture was shaken at 180 rpm and 30 °C, during which samples were withdrawn at regular intervals and extracted with an equal volume of ethyl acetate. The progress of the reaction was monitored by HPLC analysis and all experiments were conducted in triplicate.

### Production of LOPA

In a mixed system with a reaction volume of 20 mL, the final concentrations of the components were as follows: 10 mM caffeic acid (with 2% (v/v) DMSO), 20 mM MgCl_2_, 500 mM NH_4_Cl, 200 mg/L purified enzyme, and 200 mM Tris–HCl buffer (pH 8.5). The reaction flask was shaken at 180 rpm and 30 °C. Samples were regularly withdrawn and analyzed by HPLC.

## Results and discussion

### Selection and grouping of key amino acids of *Ec*mal

**Fig. 1:**
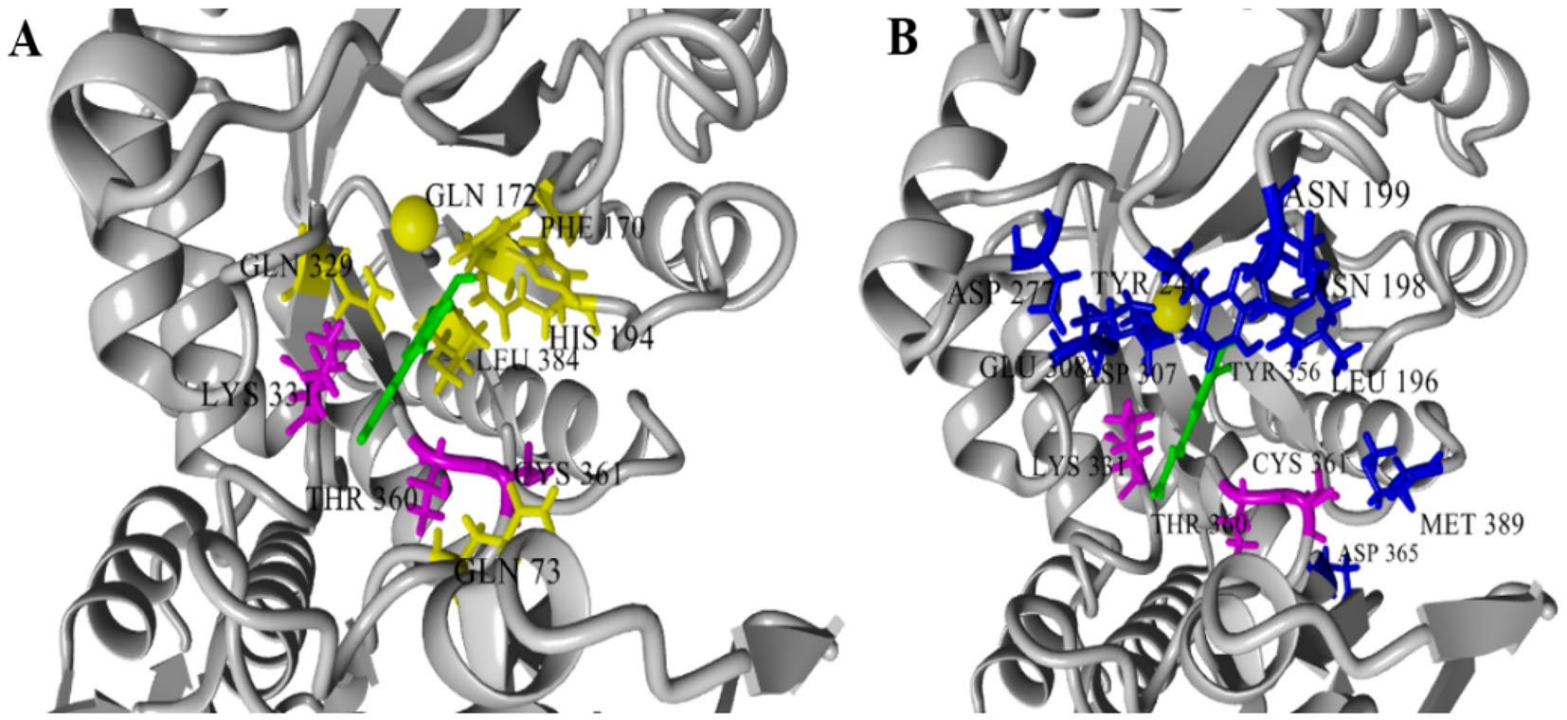
*Ec*MAL residues chosen for saturation mutagenesis marked in the homology model built by the crystal structure of MAL (PDB: 1KKR). **A** Active site mutation sites (yellow) selected on the basis of induced fit docking of amine 1 (green). **B** Residues surrounding the substrate access tunnel likewise chosen for mutagenesis (blue). The above residues were obtained through the molecular docking of Yasara and the channel recognition function of CAVER 3.0, respectively

Based on our previous studies, we found that *Ec*MAL could accept a range of short-chain unsaturated carboxylic acids, including fumaric acid, mesaconic acid, and itaconic acid, for the asymmetric synthesis of unnatural amino acids (Ni et al. 2020). However, *Ec*MAL showed low activity in the catalysis of bulky unsaturated carboxylic acids. This difference prompted us to investigate the catalytic mechanism of *Ec*MAL from a structural perspective, with a view to further improve the catalytic activity and stereoselectivity toward bulky substrates. We chose caffeic acid (**1a**) as the model compound for assessing the activity of *Ec*MAL mutants, the basic reaction of which is shown in Additional file 1: Scheme S1. Four aromatic unsaturated carboxylic acids (cinnamic acid (**1b**), *p*-hydroxycinnamic acid (**1c**), methylcinnamic acid (**1d**), and acrylic acid (**1e**)) with different substituents were also selected. These bulky compounds were selected as substrates because their amination products are important components or building blocks of therapeutic drugs, such as the Parkinson’s curative l-dopa. Considering that polyphenols can undergo a rapid color-forming reaction under the action of tyrosinase, we selected **1a** as the screening substrate to assess the efficacies of the enzyme variants. Based on the homologous three-dimensional model of *Ec*MAL, Yasara and CAVER 3.0 were used for molecular docking and substrate tunnel identification, respectively (Liu et al. 2019; Heath et al. 2014). The substrate-binding pocket and substrate tunnel have been demonstrated to have varying degrees of impact on the properties of the enzyme, thereby enhancing the differentiation of directed evolution (De Raffele et al. 2020). We simultaneously considered two groups of residues, those lining the binding pocket (73, 170, 172, 194, 329, 384) and those surrounding the substrate tunnel (196, 198, 199, 240, 277, 307, 308, 356, 365, 389). Three residues (331, 360, 361) participating in both regions were also taken into account (Fig. 1).

**Fig. 2:**
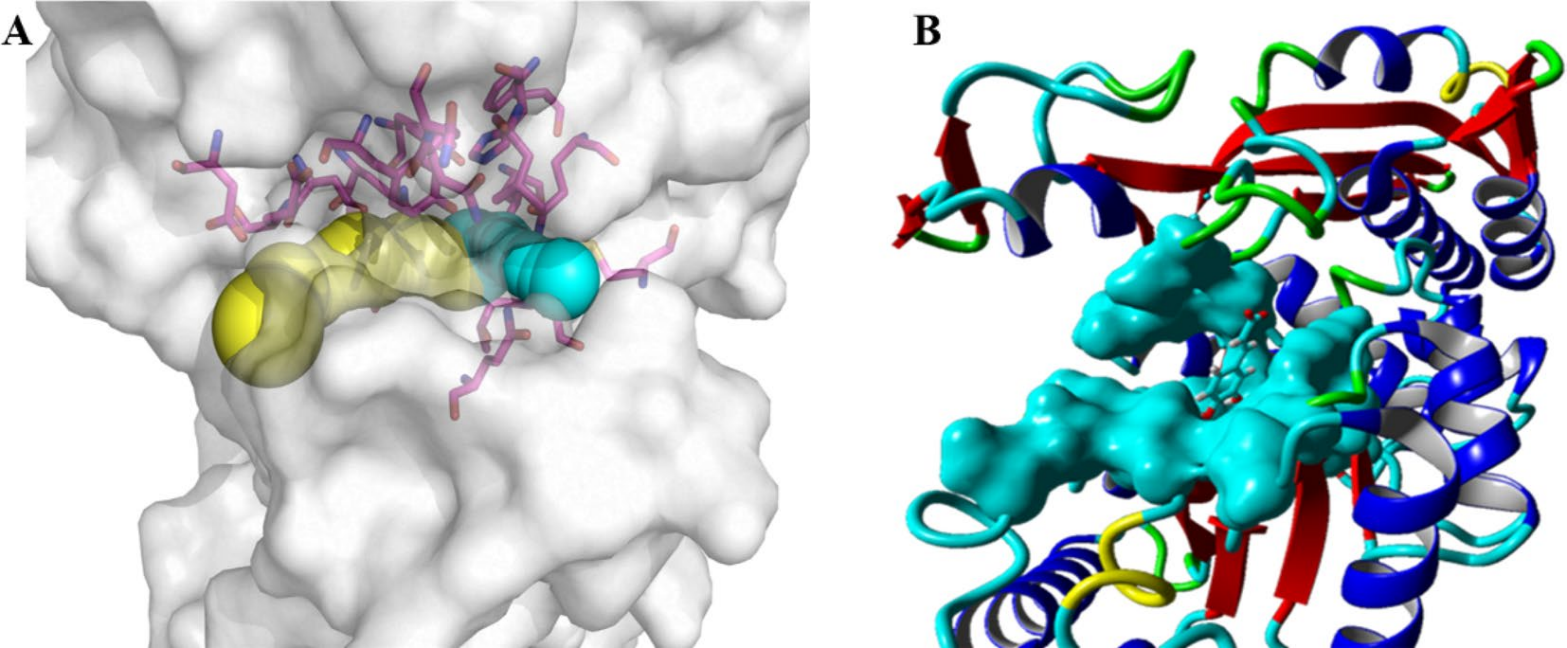
Spatial structure information of substrate–enzyme docking. **A** The position relationship between *Ec*MAL channels and the active sites. The target residues of libraries A–E are displayed in purple sticks. The substrate tunnel is shown in yellow and blue. **B** Poses of caffeic acid docked in the modeled structure of *Ec*MAL. The substrate caffeic acid is shown in azure and the selected residues envelop the substrate in a surface view. The visualization of *Ec*MAL channel, substrate and target amino acid residues was achieved by Pymol and Yasara

According to the type and spatial location of the 19 selected residues, we attempted to divide them into five groups, as shown in Fig. 2 and Table 1. Although the use of degenerate NNK codons (N = A, G, C, T; K = G) for saturation mutation would fully cover all possible amino acids, it is also accompanied by a dramatic increase in the number of screening (Wittmann et al. 2020). The degenerate NDT codons (D = A, G, T) encoding the 12 different types of amino acids (C/F/H/D/N/S/I/G/V/L/Y/R) better meet the requirements of a “smart” library (Reetz et al. 2006). Thus, NDT was considered as a simplified codon for library construction. Because the combination of simultaneous mutations increases exponentially, it is still necessary to screen at least 10^4^ levels to achieve the theoretical 95% coverage (Wang et al. 2017). On this basis, we developed a solid-phase screening method that could rapidly screen 10^3^ level transformants through color changes on a single plate. The single colonies that met the requirements were subsequently selected for further activity testing (Tang et al. 2018).

**Table 1:**
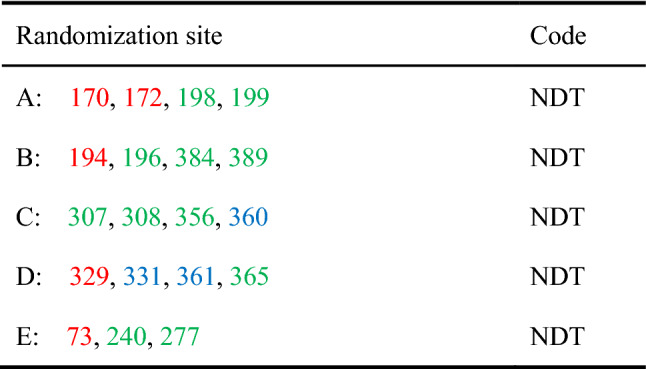
Grouping of the 19 chosen *Ec*MAL residues into five randomization sites (A, B, C, D and E) and the NDT codes used in saturation mutagenesis

The red numbers denote the “active site” positions, the green ones as the “tunnel” positions, and blue ones as the “shared” positions

### Screening and identification of active *Ec*mal variants

First of all, we designed mutation primers according to different mutation groups, and constructed the mutant libraries after amplification (Additional file 1: Figure S1). Using the solid-phase color screening procedure described in the experimental part, we performed high-throughput screening of five mutant libraries (Additional file 1: Figure S2). In the first round of screening, several mutants with activity to substrate **1a** were screened from libraries A to E, and the location and type of mutations were further determined by sequencing. The screening results are listed in Table 2 (M1–M7). Clearly, although the double mutations (M1–M3) showed new trace activity toward caffeic acid compared with the wild type, its activity and stereoselectivity were far from the expectations (Li et al. 2019). However, the coincidence that the effective mutants were concentrated in libraries B, D, and E caught our attention. The triple mutants (M4–M6) screened by these libraries further indicated that the selected hot sites in libraries B, D, and E had a great influence on the enzyme to accept bulky substrates. In particular, a quadruple mutation M7 (Q329F/K331R/Y356S/C361L) suddenly exhibited a mutation transition effect, with remarkable improvements in catalytic activity and selectivity. Motivated by these results, M7 was chosen as the parental enzyme for further iterative combinations. Subsequently, a second round of screening experiments were implemented for libraries B and E using M7 as the template. After screening approximately 5000 clones and confirming the specific activities, we identified the best variants (M8) toward the model substrates, which are shown in Table 2. Compared with the catalytic performance identified by other mutants, the M8 presented about 15-fold improvement and the selectivity was also greatly improved. Considering that M8 was created by partial iterative mutation of M7, and the catalytic activity was improved while the stereoselectivity was maintained, the results indicate a prominent synergistic influence among these six residues.

**Table 2:** Properties of the *Ec*MAL variants

Enzyme and mutants	Mutation sites	Specific activity (U/g_protein_)^a^	% ee^b^
WT *Ec*MAL	None	nd^c^	nd^c^
M1	Q329S/K331R	5.4 ± 0.5	16 (S)
M2	Q73F/Y240C	4.4 ± 0.4	4.4 (S)
M3	F170L/E172F	2.5 ± 0.5	39 (S)
M4	Q329S/K331G/C361C	6.2 ± 0.8	86(S)
M5	Q73V/Y240C/D277C	7.3 ± 0.1	28(S)
M6	F170S/E172R/D307L	9.0 ± 0.3	79 (S)
M7	Q329F/K331R/Y356S/C361L	26.2 ± 0.2	99 (S)
M8	E172R/Y240S/D307L/Q329F/Y356S/C361L	38.6 ± 0.5	99 (S)

^a^Specific activity was determined at pH 8.0 and 30 °C using purified enzyme and omeprazole sulfide

^b^The ee values were determined by HPLC

^c^Not detected

^d^The bold mutations indicate newly involved mutation(s) in each round

**Fig. 3:**
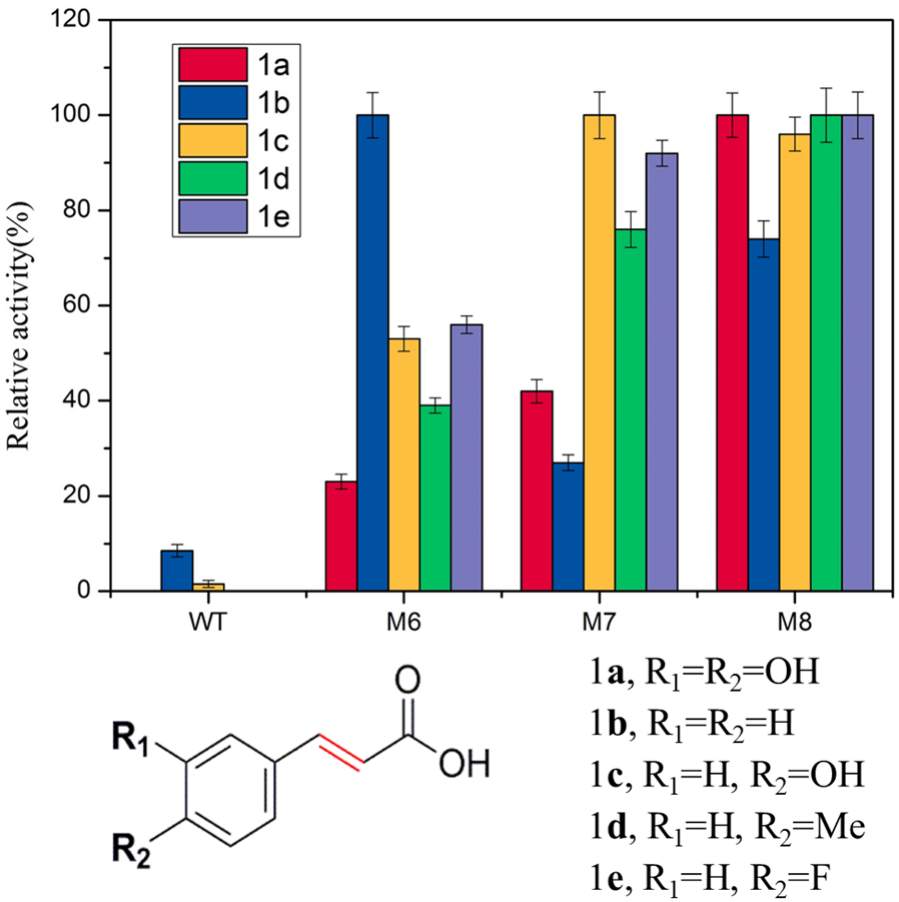
Activity fingerprints of *Ec*MAL and its variants (M6–M8) with different substrates. The activity was measured by HPLC, and relative activity is measured as a percentage of the optimum activity of each substrate (**1a** caffeic acid, **1b** cinnamic acid, **1c** *p*-hydroxycinnamic acid, **1d** methylcinnamic acid, **1e** acrylic acid)

To fully investigate the catalytic performance of the selected mutants, several substrates with varying sizes and structures, including **1a**, **1b**, **1c**, **1d**, and **1e**, were selected as candidates for the activity testing of WT and several representative mutants. Notably, there was trace or even no specific activity observed for the WT. Moreover, compared with the bulky caffeic acid, different mutants exhibited obviously different catalytic activities (Ni et al. 2020). Interestingly, the triple site mutant M6 showed significant improvement in activity toward **1b** and was even better than M8 (Fig. 3). The M8 mutant also obviously shows a great advantage in accepting other substrates with substituents. These results indicated that the optimal substrate of *Ec*MAL appears at each stage of evolutionary route and the possibility of developing in different catalytic directions.

### Structure and computational simulation analysis of *Ec*MAL variants

**Fig. 4:**
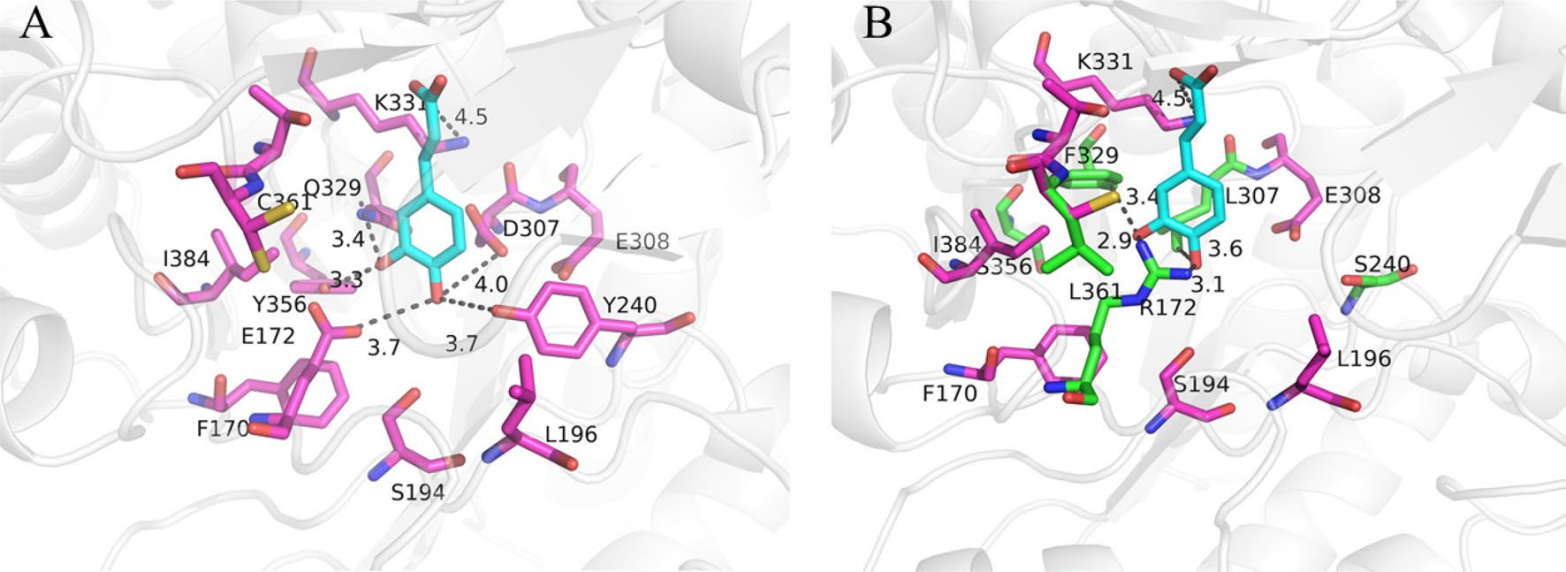
Comparison of hydrogen bonding in *Ec*MAL_WT_ (**A**) and its variant *Ec*MAL_M8_ (**B**). The docking substrate (**1a**) and amino acid residues are shown in sticks. The hydrogen bonds are shown in black imaginary lines

To gain insight into the structure–activity relationship of the dramatically altered substrate specificity, the three-dimensional structures of *Ec*MAL_WT_ and EcMAL_M8_ were first compared by Yasara. As shown in Fig. 4, three polar mutant residues near the active center were replaced with non-polar residues (Q172R, Q329F, C361L) in EcMAL_M8_, which increased the hydrophobic interaction between the active site and the substrate. Moreover, the residues H194, Q329 and K331 form the catalytic triad (Teze et al. 2020). Among them, the E329 responsible for stabilizing the enolate anion was replaced with phenylalanine, which increased the pi–pi stacked interaction with the substrate. Because the catalytic active center of *Ec*MAL is a wide crack shape, there is theoretically no situation in which the substrate has difficulty in contacting the active center because of the spatial resistance. Therefore, it is speculated that the critical factor affecting the enzyme catalytic activity is the unstable binding between the substrate and enzyme. The docking of the mutant and the substrate also confirmed this view, as the catalytic pocket of the enzyme was more accessible to the substrate.

To investigate the conformational changes of the catalytic active site, whole systems were equilibrated by MD at 10 ns without any restriction (Teze et al. 2020). Our goal is to identify conformational changes that regulate the accessibility of the active sites. Based on the snapshots of the MD simulations, it is easy to observe that because of the substitution of phenylalanine at position 329, the enhancement in electrostatic attraction to aromatic substrates makes it more accessible to the binding domain. The average per residue root mean square fluctuation (RMSF) of the Cα atoms of a monomer in *Ec*MAL was calculated as a measurement of protein flexibility (Additional file 1: Figure S3). Residues L384 and M389 near the tunnel in the *Ec*MAL_WT_ exhibited lower flexibility, which was unfavorable to the bulky substrate in and out of the tunnel. The RMSF changes of the mutant residues also supported this hypothesis.

### Catalytic performance and substrate specificity of *Ec*mal variants

**Fig. 5:**
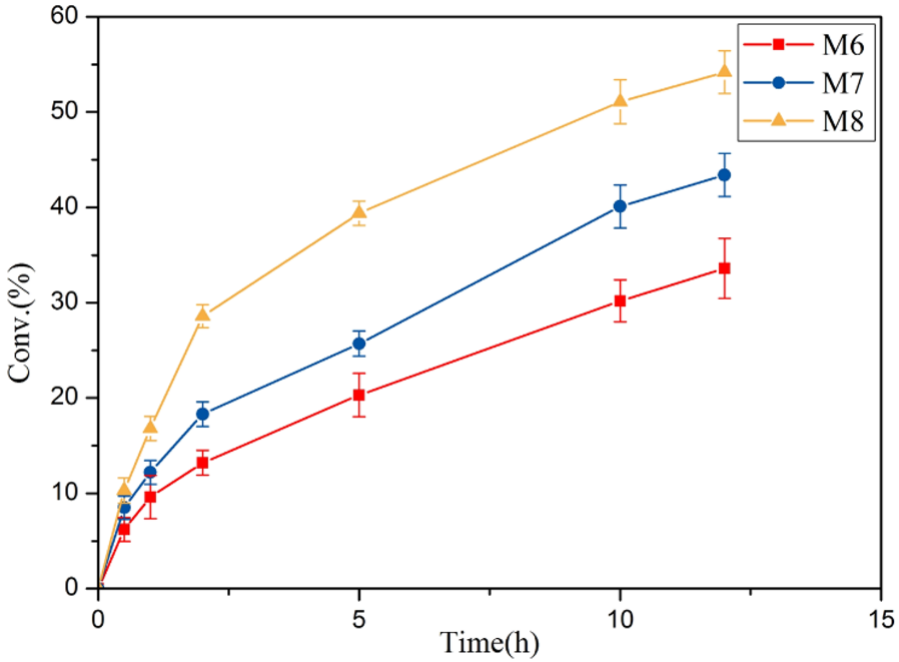
Progress curves of caffeic acid catalyzed by the purified enzymes of three *Ec*MAL variants (M6, M7, M8). The enzymatic amination addition of caffeic acid (10 mM) was performed at 30 °C and pH 8.5 with a dose of each 0.5 mg/mL purified enzymes under the same conditions

We subsequently determined the catalytic performance of *Ec*MAL variants for L-dopa synthesis. The engineered *Ec*MALs were used to react under optimal conditions. As shown in Fig. 5, the hydroamination addition reaction catalyzed by variant M8 reached a conversion rate higher than 50% within 12 h. Meanwhile, it was also significantly improved compared to other mutants. To further evaluate the specificity responsible for different substrates in *Ec*MAL variants, the kinetic parameters of M6–M8 with substrates **1a**–**1b** were also considered (Table 3). M8 was obtained from M7 through the iterative mutation of two residues 73 and 389. However, it is economical to achieve a large increase in the turnover rate (Kcat from 36.8 to 46.8) with a small sacrifice of affinity (Km from 1.25 to 1.32 mM) for substrate **1a**. Instead, the best variant for **1a** did not exert higher activity toward **1b**, which was equipped with a relatively smaller substituent. M6 showed strong activity with the lowest Km (1.23 mM) observed. This may be due to the different epistatic effects of different residues, which simultaneous mutant near the active center and channel of *Ec*MAL, resulting in different non-covalent forces on different substrates.

**Table 3:** Kinetic characterization of the *Ec*MAL variants M6–M8 to substrates **1a** and **1b**

Enzyme and mutants^a^	Substrate	*K*_m_ (mM)	*K*_cat_ (min^−1^)	*K*_cat_/*K*_m_ (min^−1^ mM^−1^)
M8	**1a**	1.32 ± 0.09	61.76 ± 0.8	46.8
M7	**1a**	1.25 ± 0.05	46.02 ± 0.4	36.8
M6	**1a**	1.47 ± 0.05	34.5 ± 0.74	23.5
M8	**1b**	1.34 ± 0.06	31.48 ± 0.63	23.5
M7	**1b**	1.96 ± 0.08	21.5 ± 0.38	11.0
M6	**1b**	1.23 ± 0.03	42.5 ± 0.72	34.6

^a^Three parallels were measured for each sample. The reaction mixture contained 500 mM Tris–HCl buffer (pH 8.5), 500 mM NH_4_Cl, 20 mM Mg^2+^, **1a**: caffeic acid (0.25–10 mM)/**1b**: cinnamic acid (0.2–10 mM)

## Conclusion

In summary, with the aim of activating *Ec*MAL for the amination of bulky unsaturated carboxylic acids, we applied structure-guided strategies for the first time to simultaneously perform saturation mutagenesis on the positions of the residues lining the binding pocket and surrounding the substrate tunnel to successfully evolve the enzyme. Iterative saturation mutagenesis has been applied to optimize the results. Using an effective solid-phase screening method based on the oxidative color-forming reaction of polyphenols by tyrosinase, the effective variants could be quickly screened. Compared with traditional chromatographic screening, this method significantly improves the screening efficiency of the mutant libraries. After several rounds of screening, a series of variants with pronounced increases in catalytic activity and stereoselectivity for bulky substrates (including caffeic acid) were obtained, achieving a substrate specificity that shifted from small to large. Subsequently, the representative mutants were selected to explore the adaptability of bulky substrates by accepting four different substituted aromatic unsaturated carboxylic acids. Meanwhile, the synthesis of levodopa, an anti-dementia drug, was further investigated. The activity of the optimal mutant (M8) to caffeic acid increased by about 15-fold, and the conversion rate was more than 50% within 12 h. To gain deeper insight, further study into the crucial residues of engineered enzymes was carried out to explore the regulation mechanism in the substrate spectrum. MD simulation analysis and tunnel prediction were used to explore the binding relationship between the substrate and enzyme in bulky substrate catalysis. We look forward to using this work as a starting point to develop more valuable enzymatic hydroamination addition reactions to achieve green and economic synthesis of unnatural amino acids.

### Supplementary Information

**Additional file 1**: **Scheme S1**. Asymmetric amination of caffeic acid by EcMAL for the production of L-dopa. **Figure S1**. Primer design and library creation of WT Ec-MAL. **Figure S2**. Schematic representation of a solid-phase screening assay. **Figure S3**. Molecular dynamics simulation results of EcMAL. **Table S1**. List of primers for mutation library construction.

## Data Availability

All data generated or analyzed during this study are included in this article.

## Abbreviations

MAL: Methylaspartate lyase
*Ec*MAL: Methylaspartate lyase from *Escherichia coli* O157:H7
AL: Ammonia lyase
l-dopa: Levodopa
DMSO: Dimethyl sulfoxide
IPTG: Isopropyl-β-*d*-thiogalactoside
MD: Molecular dynamics
RMSF: Root mean square fluctuation
