# Supplementary material for: Structure-guided protein engineering of ammonia lyase for efficient synthesis of sterically bulky unnatural amino acids
Source: Bioresour Bioprocess. 2021 Oct 19;8(1):103. doi: 10.1186/s40643-021-00456-5 (PMC10992142; doi:10.1186/s40643-021-00456-5)
Supplement: Supplementary file 1 — Additional file 1: Scheme S1. Asymmetric amination of caffeic acid by EcMAL for the production of L-dopa. Figure S1. Primer design and library creation of WT Ec-MAL. Figure S2. Schematic representation of a solid-phase screening assay. Figure S3. Molecular dynamics simulation results of EcMAL. Table S1. List of primers for mutation library construction. [file 40643_2021_456_MOESM1_ESM.docx]

**Additional file 1**

**Structure-based protein engineering of ammonia lyase for efficient synthesis of sterically bulky unnatural amino acids**

Zi-Fu Ni, Pei Xu, Min-Hua Zong, Wen-Yong Lou^*^

Laboratory of Applied Biocatalysis, School of Food Science and Engineering, South China University of Technology, No. 381 Wushan Road, Guangzhou 510640, Guangdong, China

^*^Corresponding author. Tel.: +86-20-22236669; fax: +86-20-22236669.

E-mail: [wylou@scut.edu.cn](mailto:wylou@scut.edu.cn)

Contents

[1.Supporting Scheme 3](#_Toc84418529)

[Scheme S1. Asymmetric amination of caffeic acid by EcMAL for the production of L-dopa. 3](#_Toc84418530)

[2.Supporting Figures 4](#_Toc84418531)

[Figure S1. Primer design and library creation of WT Ec-MAL. 4](#_Toc84418532)

[Figure S2. Schematic representation of a solid-phase screening assay. 5](#_Toc84418533)

[Figure S3. Molecular dynamics simulation results of EcMAL 6](#_Toc84418534)

[2. Supporting Tables 7](#_Toc84418535)

[Table S1. List of primers for mutation library construction 7](#_Toc84418536)

[3. References: 8](#_Toc84418537)

# 1.Supporting Scheme

## Scheme S1. Asymmetric amination of caffeic acid by *Ec*MAL for the production of L-dopa.

# 2.Supporting Figures

##
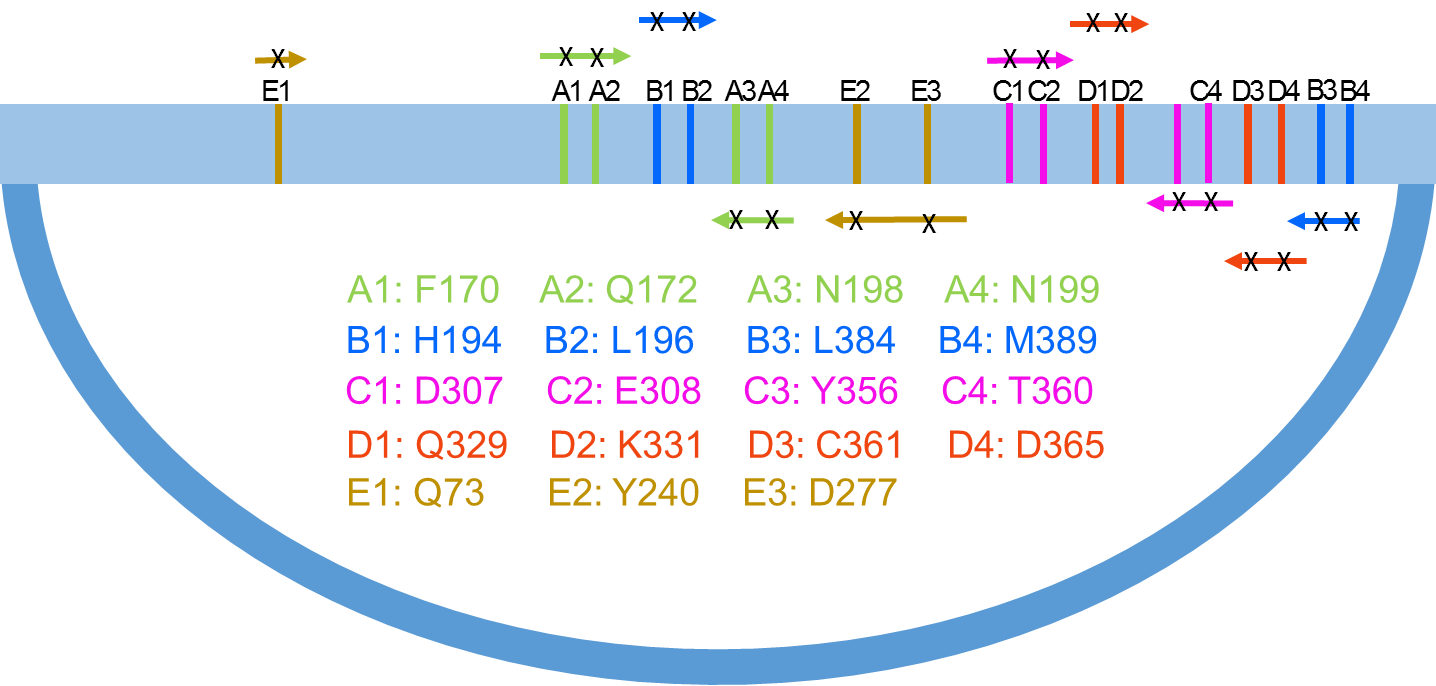
 Figure S1. Primer design and library creation of WT *Ec*-MAL.

NDT were used as the building blocks. Megaprimer were used to synthesize the whole mutant plasmid.


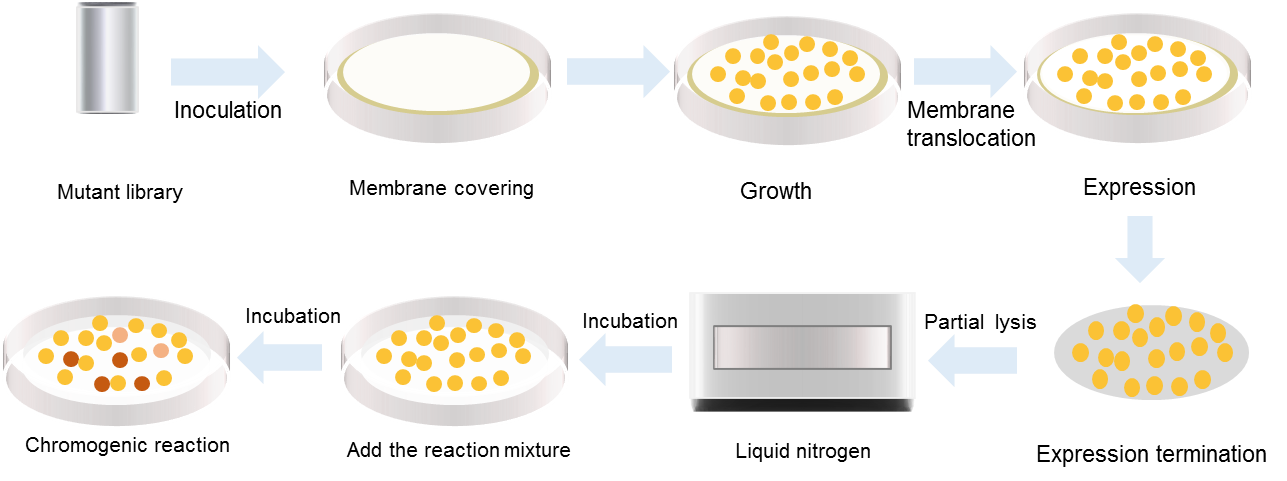


## Figure S2. Schematic representation of a solid-phase screening assay.

Nylon membranes loaded on LB agar plates were inoculated with Escherichia coli cells expressing *Ec*MAL variants. After growth and protein production, membranes were transferred into a reaction mixture solution, and colonies expressing active variants were identified by a red colony.


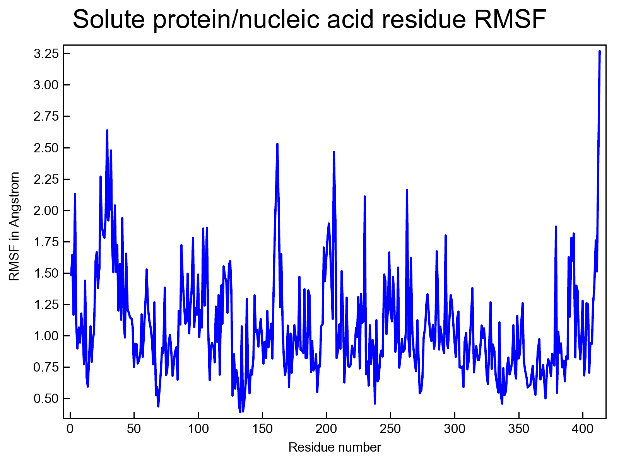

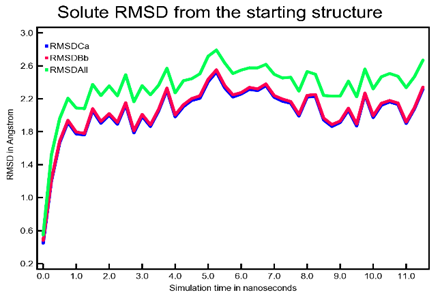


## Figure S3. Molecular dynamics simulation results of *Ec*MAL

# 2. Supporting Tables

Table S1. List of primers for mutation library construction

| Number | Sequence(5’to 3’) |
| --- | --- |
| 170/172-NDT-F | TCCATTCCATTANDTGGTNDTAGCGGCGACG |
| 170/172-NDT-R | ATCGTCGCCGCTAHNACCAHNTAATGGAATG |
| 198/199-NDT-F | CATGCGCTGATTNDTNDTGTCGAAGAGA |
| 198/199-NDT-R | CTTCTCTTCGACAHNAHNAATCAGCGCA |
| 194/196-NDT-F | GACGTGCTGCCCNDTGCGNDTATTAATA |
| 194/196-NDT-R | GTTATTAATAHNCGCAHNGGGCAGCACG |
| 240-NDT-F | CACATCGATGTANDTGGCACTATCG |
| 240-NDT-R | ACCGATAGTGCCAHNTACATCGATG |
| 277-NDT-F | GAAGGGCCGGTCNDTGCCGGTAACA |
| 277-NDT-R | CTTGTTACCGGCAHNGACCGGCCCT |
| 307/308-NDT-F | AAAATTGTGGCCNDTNDTTGGTGTAACA |
| 307/308-NDT-R | GGTGTTACACCAAHNAHNGGCCACAAT |
| 356/360-NDT-F | TGGAAGCGNDTCAGGGCGGCNDTTGCAATG |
| 356/360-NDT-R | TTCATTGCAAHNGCCGCCCTGAHNCGCTTC |
| 361/365-NDT-F | GGCGGCACCNDTAATGAAACTNDTGTCAGTG |
| 361/365-NDT-R | GGCACTGACAHNAGTTTCATTAHNGGTGCCG |
| 329/331-NDT-F | TGTCACATGGTGNDTATCNDTACCCCGG |
| 329/331-NDT-R | ATCCGGGGTAHNGATAHNCACCATGTGA |
| 73-NDT-F | GACTGTACTGCCGTANDTTATTCCG |
| 73-NDT-R | CCCGGAATAAHNTACGGCAGTACAG |
| 384/389-NDT-F | CCCATGCGTATGNDTGTAAAACCAGGGNDTGGCTTTG |
| 384/389-NDT-R | GTCAAAGCCAHNCCCTGGTTTTACAHNCATACGCATG |

*The mutation sites were underlined.

# 3. References:

(1) Lambrughi, M.; Maršić, Ž. S.; Saez-Jimenez, V.; Mapelli, V.; Olsson, L.; Papaleo, E. Conformational Gating in Ammonia Lyases. *bioRxiv* **2019**. https://doi.org/10.1101/583088.

(2) Cheng, X.; Chen, X.; Feng, J.; Wu, Q.; Zhu, D. Structure-Guided Engineering of: Meso -Diaminopimelate Dehydrogenase for Enantioselective Reductive Amination of Sterically Bulky 2-Keto Acids. *Catal. Sci. Technol.* **2018**, *8* (19), 4994–5002. https://doi.org/10.1039/c8cy01426d.

(3) Yuan, S.; Chan, H. C. S.; Filipek, S.; Vogel, H. PyMOL and Inkscape Bridge the Data and the Data Visualization. *Structure*. 2016. https://doi.org/10.1016/j.str.2016.11.012.

(4) Yu, S.; Yao, P.; Li, J.; Feng, J.; Wu, Q.; Zhu, D. Improving the Catalytic Efficiency and Stereoselectivity of a Nitrilase from: Synechocystis Sp. PCC6803 by Semi-Rational Engineering En Route to Chiral γ-Amino Acids. *Catal. Sci. Technol.* **2019**, *9* (6), 1504–1510. https://doi.org/10.1039/c8cy02455c.

(5) Zhang, Y.; Wu, Y. Q.; Xu, N.; Zhao, Q.; Yu, H. L.; Xu, J. H. Engineering of Cyclohexanone Monooxygenase for the Enantioselective Synthesis of (S)-Omeprazole. *ACS Sustain. Chem. Eng.* **2019**, *7* (7), 7218–7226. https://doi.org/10.1021/acssuschemeng.9b00224.

(6) Wang, J. B.; Lonsdale, R.; Reetz, M. T. Exploring Substrate Scope and Stereoselectivity of P450 Peroxygenase OleTJE in Olefin-Forming Oxidative Decarboxylation. *Chem. Commun.* **2016**, *52* (52), 8131–8133. https://doi.org/10.1039/c6cc04345c.
